# Supplementary material for: Unveiling the Bioactive Potential of the Invasive Jellyfish Phyllorhiza punctata Through Integrative Transcriptomic and Proteomic Analyses
Source: Biomolecules. 2025 Aug 4;15(8):1121. doi: 10.3390/biom15081121 (PMC12383608; doi:10.3390/biom15081121)
Supplement: Supplementary file 1 [file biomolecules-15-01121-s001.zip › Suplementary Material/FileS3/PEPFOLD4/XP_065055677_4807/clusters.html]

 PEP-FOLD clusters


Download archive of all models  here   

|  |  |  |
| --- | --- | --- |
| # | file | sOPEP |
| # cluster 1 - Size: 15 |  |  |
|  | XP\_065055677\_4807-model1 | -9.95258 |
|  | XP\_065055677\_4807-model1.1 | -9.95258 |
|  | XP\_065055677\_4807-model1.2 | -9.95258 |
|  | XP\_065055677\_4807-model1.3 | -9.95258 |
|  | XP\_065055677\_4807-model1.4 | -9.95258 |
|  | XP\_065055677\_4807-model1.5 | -9.95258 |
|  | XP\_065055677\_4807-model1.6 | -9.95258 |
|  | XP\_065055677\_4807-model1.7 | -9.95258 |
|  | XP\_065055677\_4807-model1.8 | -9.95258 |
|  | XP\_065055677\_4807-model1.9 | -9.95258 |
|  | XP\_065055677\_4807-model1.10 | -9.95258 |
|  | XP\_065055677\_4807-model1.11 | -9.95258 |
|  | XP\_065055677\_4807-model1.12 | -9.95258 |
|  | XP\_065055677\_4807-model1.13 | -9.95258 |
|  | XP\_065055677\_4807-model1.14 | -9.95258 |
| # cluster 2 - Size: 35 |  |  |
|  | XP\_065055677\_4807-model2 | -9.94387 |
|  | XP\_065055677\_4807-model2.1 | -9.94387 |
|  | XP\_065055677\_4807-model2.2 | -9.94387 |
|  | XP\_065055677\_4807-model2.3 | -9.94387 |
|  | XP\_065055677\_4807-model2.4 | -9.94387 |
|  | XP\_065055677\_4807-model2.5 | -9.94387 |
|  | XP\_065055677\_4807-model2.6 | -9.94387 |
|  | XP\_065055677\_4807-model2.7 | -9.94387 |
|  | XP\_065055677\_4807-model2.8 | -9.94387 |
|  | XP\_065055677\_4807-model2.9 | -9.94387 |
|  | XP\_065055677\_4807-model2.10 | -9.94387 |
|  | XP\_065055677\_4807-model2.11 | -9.94387 |
|  | XP\_065055677\_4807-model2.12 | -9.94387 |
|  | XP\_065055677\_4807-model2.13 | -9.94387 |
|  | XP\_065055677\_4807-model2.14 | -9.94387 |
|  | XP\_065055677\_4807-model2.15 | -9.94387 |
|  | XP\_065055677\_4807-model2.16 | -9.94387 |
|  | XP\_065055677\_4807-model2.17 | -9.94387 |
|  | XP\_065055677\_4807-model2.18 | -9.94387 |
|  | XP\_065055677\_4807-model2.19 | -9.94387 |
|  | XP\_065055677\_4807-model2.20 | -9.94387 |
|  | XP\_065055677\_4807-model2.21 | -9.94387 |
|  | XP\_065055677\_4807-model2.22 | -9.94387 |
|  | XP\_065055677\_4807-model2.23 | -9.94387 |
|  | XP\_065055677\_4807-model2.24 | -9.94387 |
|  | XP\_065055677\_4807-model2.25 | -9.94387 |
|  | XP\_065055677\_4807-model2.26 | -9.94387 |
|  | XP\_065055677\_4807-model2.27 | -9.94387 |
|  | XP\_065055677\_4807-model2.28 | -9.94387 |
|  | XP\_065055677\_4807-model2.29 | -9.94387 |
|  | XP\_065055677\_4807-model2.30 | -9.94387 |
|  | XP\_065055677\_4807-model2.31 | -9.94387 |
|  | XP\_065055677\_4807-model2.32 | -9.94387 |
|  | XP\_065055677\_4807-model2.33 | -9.94387 |
|  | XP\_065055677\_4807-model2.34 | -9.94387 |
| # cluster 3 - Size: 29 |  |  |
|  | XP\_065055677\_4807-model3 | -9.89638 |
|  | XP\_065055677\_4807-model3.1 | -9.89638 |
|  | XP\_065055677\_4807-model3.2 | -9.89638 |
|  | XP\_065055677\_4807-model3.3 | -9.89638 |
|  | XP\_065055677\_4807-model3.4 | -9.89638 |
|  | XP\_065055677\_4807-model3.5 | -9.89638 |
|  | XP\_065055677\_4807-model3.6 | -9.89638 |
|  | XP\_065055677\_4807-model3.7 | -9.89638 |
|  | XP\_065055677\_4807-model3.8 | -9.89638 |
|  | XP\_065055677\_4807-model3.9 | -9.89638 |
|  | XP\_065055677\_4807-model3.10 | -9.89638 |
|  | XP\_065055677\_4807-model3.11 | -9.89638 |
|  | XP\_065055677\_4807-model3.12 | -9.89638 |
|  | XP\_065055677\_4807-model3.13 | -9.89638 |
|  | XP\_065055677\_4807-model3.14 | -9.89638 |
|  | XP\_065055677\_4807-model3.15 | -9.89638 |
|  | XP\_065055677\_4807-model3.16 | -9.89638 |
|  | XP\_065055677\_4807-model3.17 | -9.89638 |
|  | XP\_065055677\_4807-model3.18 | -9.89638 |
|  | XP\_065055677\_4807-model3.19 | -9.89638 |
|  | XP\_065055677\_4807-model3.20 | -9.89638 |
|  | XP\_065055677\_4807-model3.21 | -9.89638 |
|  | XP\_065055677\_4807-model3.22 | -9.89638 |
|  | XP\_065055677\_4807-model3.23 | -9.89638 |
|  | XP\_065055677\_4807-model3.24 | -9.89638 |
|  | XP\_065055677\_4807-model3.25 | -9.89638 |
|  | XP\_065055677\_4807-model3.26 | -9.89638 |
|  | XP\_065055677\_4807-model3.27 | -9.89638 |
|  | XP\_065055677\_4807-model3.28 | -9.89638 |
| # cluster 4 - Size: 18 |  |  |
|  | XP\_065055677\_4807-model4 | -9.88467 |
|  | XP\_065055677\_4807-model4.1 | -9.88467 |
|  | XP\_065055677\_4807-model4.2 | -9.88467 |
|  | XP\_065055677\_4807-model4.3 | -9.88467 |
|  | XP\_065055677\_4807-model4.4 | -9.88467 |
|  | XP\_065055677\_4807-model4.5 | -9.88467 |
|  | XP\_065055677\_4807-model4.6 | -9.88467 |
|  | XP\_065055677\_4807-model4.7 | -9.88467 |
|  | XP\_065055677\_4807-model4.8 | -9.88467 |
|  | XP\_065055677\_4807-model4.9 | -9.88467 |
|  | XP\_065055677\_4807-model4.10 | -9.88467 |
|  | XP\_065055677\_4807-model4.11 | -9.88467 |
|  | XP\_065055677\_4807-model4.12 | -9.88467 |
|  | XP\_065055677\_4807-model4.13 | -9.88467 |
|  | XP\_065055677\_4807-model4.14 | -9.88467 |
|  | XP\_065055677\_4807-model4.15 | -9.88467 |
|  | XP\_065055677\_4807-model4.16 | -9.88467 |
|  | XP\_065055677\_4807-model4.17 | -9.88467 |
| # cluster 5 - Size: 3 |  |  |
|  | XP\_065055677\_4807-model5 | -9.87917 |
|  | XP\_065055677\_4807-model5.1 | -9.87917 |
|  | XP\_065055677\_4807-model5.2 | -9.87917 |
|  |  |  |
